# An Incessant Tachycardia: What Is the Mechanism?

**DOI:** 10.19102/icrm.2021.120407

**Published:** 2021-04-15

**Authors:** Matthew J. Singleton, Jayanthi N. Koneru, Prashant D. Bhave, S. Patrick Whalen

**Affiliations:** ^1^Section of Cardiology, Department of Internal Medicine, Wake Forest School of Medicine, Winston-Salem, NC, USA; ^2^Division of Cardiology, Department of Internal Medicine, Virginia Commonwealth University School of Medicine, Richmond, VI, USA

**Keywords:** Activation mapping, orthodromic reciprocating tachycardia, permanent junctional reciprocating tachycardia, radiofrequency ablation

## Abstract

A 39-year-old man presented with lifelong palpitations, a mildly reduced left ventricular ejection fraction, and incessant tachycardia. Electrocardiography revealed a regular, one-to-one supraventricular tachycardia with superiorly directed P-waves and a long R–P interval. The differential diagnosis of the tachycardia, response to invasive electrophysiologic maneuvers, and treatment with catheter ablation are discussed.

## Case presentation

**Figure 1:**
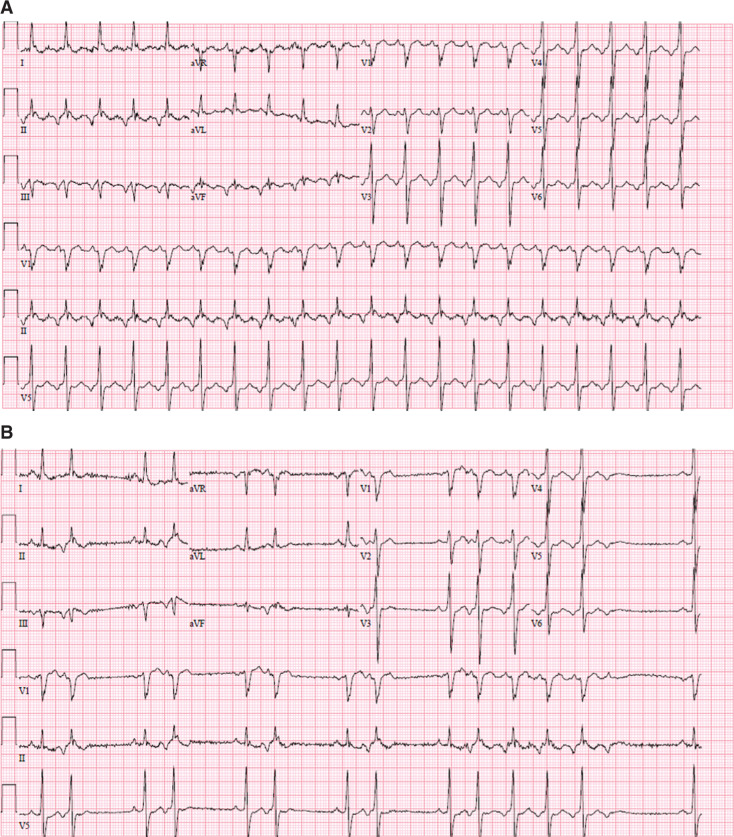
Presenting 12-lead ECGs. **A:** A regular, one-to-one supraventricular tachycardia with superiorly-directed P-waves and a long-R–P interval. **B:** Premature atrial beats at a fixed coupling interval with P-wave morphology similar to that seen during tachycardia; induction of tachycardia in sinus rhythm; variability in ventriculoatrial time, which prolongs over the course of the run of nonsustained tachycardia; and termination with an atrial depolarization.

**Figure 2:**
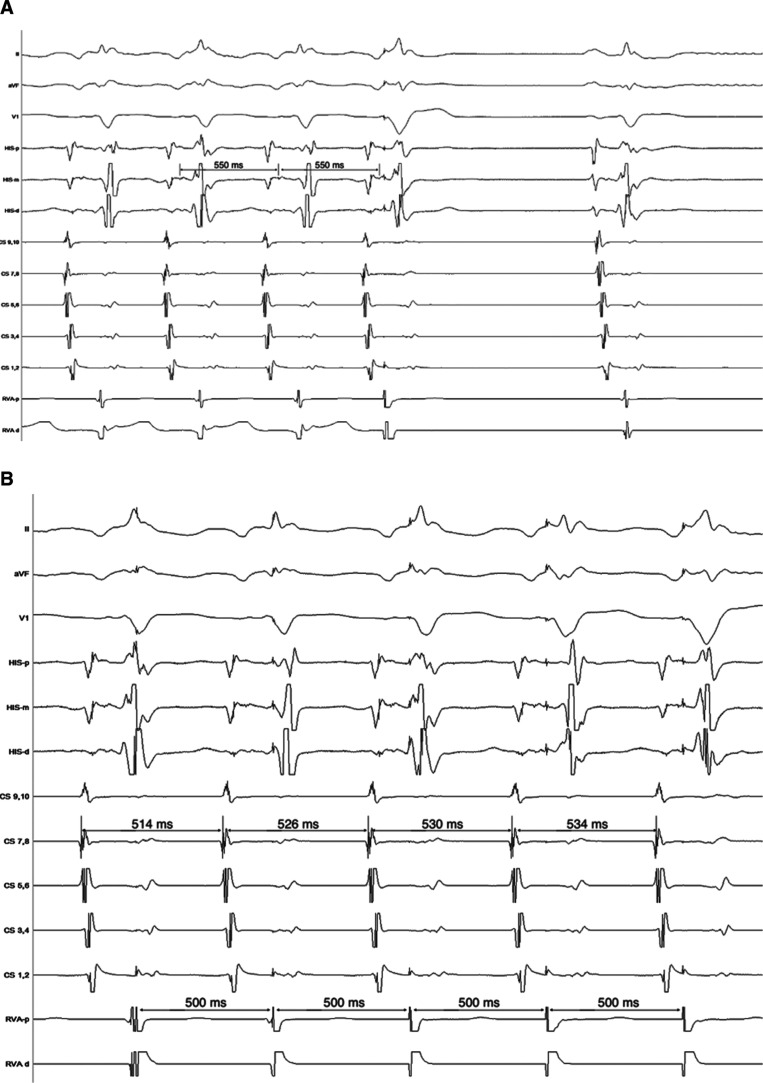
**A:** Response to His-refractory premature ventricular depolarization. **B:** Response to ventricular overdrive pacing.

A 39-year-old man was seen in consultation for the evaluation of longstanding palpitations. His presenting 12-lead electrocardiograms (ECGs) are provided in **Figure 1** and revealed a mildly reduced left ventricular ejection fraction. He was referred for diagnostic electrophysiology study. At baseline, his tachycardia was nearly incessant and easily inducible with atrial and ventricular stimulation. His-refractory ventricular premature depolarizations (VPDs) and ventricular overdrive pacing elicited the responses shown in **Figure 2**. Based on the tracings, what is the mechanism of the tachycardia?

## Discussion

The first ECG shows a regular, one-to-one supraventricular tachycardia with superiorly-directed P-waves and a long-R–P interval **(Figure 1A)**. The differential diagnosis includes atypical (fast–slow) atrioventricular nodal reentrant tachycardia (AVNRT), atrial tachycardia with a low-atrial site of origin, and orthodromic reciprocating tachycardia (ORT) with a slowly conducting accessory pathway. The second ECG **(Figure 1B)** demonstrates premature atrial beats at a fixed coupling interval with P-wave morphology similar to that seen during tachycardia; induction of tachycardia in sinus rhythm; variability in ventriculoatrial time, which prolongs over the course of the run of nonsustained tachycardia; and termination with an atrial depolarization (**Figure 1**, bottom).

Termination with an atrial depolarization makes atrial tachycardia unlikely, as this observation would require fortuitous tachycardia termination and block in the AV node simultaneously. The progressively increasing ventroculoatrial time over the course of the run of nonsustained tachycardia suggests decremental conduction in the retrograde limb of the circuit. Initiation of tachycardia during sinus rhythm, coupled with the incessant nature of the tachycardia, suggests a wide excitable gap for induction. In aggregate, these ECG findings are suggestive of ORT using a slowly conducting pathway as the retrograde limb of the circuit, known as permanent junctional reciprocating tachycardia (PJRT).

Diagnostic electrophysiology study demonstrated that a His-refractory VPD terminated the tachycardia without activation of the atrium **(Figure 2A)**, which is inconsistent with atypical AVNRT, as a His-refractory VPD should not have access to the circuit in atypical AVNRT in the absence of a bystander nodofascicular pathway.^1^ Progressively premature activation of the right ventricle via a series of consecutive His-refractory VPDs delays the subsequent atrial depolarization, demonstrating decremental conduction **(Figure 2B)**.^2,3^ The fact that the next atrial beat is delayed by VPDs proves not just the presence but also the participation of the pathway in the tachycardia circuit. Similarly, reproducible termination of the tachycardia by His-refractory VPDs without activation of the atria proves participation of the pathway.

**Figure 3:**
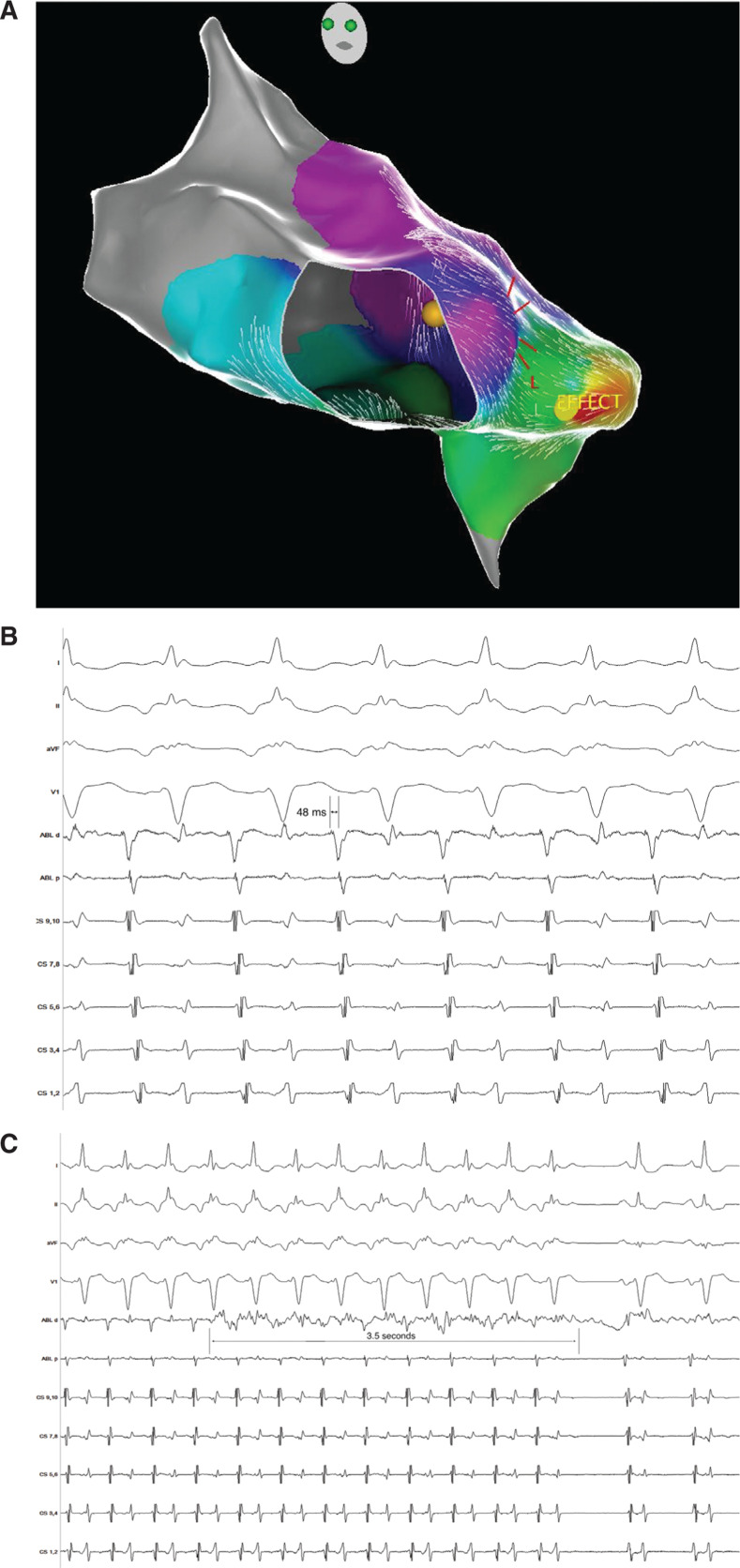
**A:** Electroanatomic map of the atrial activation sequence, demonstrating atrial insertion of the pathway just anterior to the coronary sinus ostium. **B:** Fractionated electrogram at the effective site preceded the initiation of the P-wave by 48 ms. **C:** Termination of the tachycardia with block in the retrograde limb after 3.5 seconds of radiofrequency energy delivery.

Activation mapping revealed an atrial insertion just anterior to the coronary sinus ostium^4^ **(Video 1)**; at this site, fractionated electrograms were found that preceded the P-wave initiation by 48 ms **(Figures 3A and 3B)**. After 3.5 seconds of radiofrequency energy delivery, the tachycardia terminated with block in the retrograde limb **(Figure 3C)**. Postablation, aggressive atrial and ventricular pacing and extrastimuli could not induce tachycardia, despite isoproterenol challenge.

In summary, ORT using a slowly conducting accessory pathway (PJRT) is characterized by (1) signature findings on the 12-lead ECG, including induction in sinus rhythm and evidence of decremental conduction; (2) conditions for reentry that are perennially present due to a wide excitable gap for induction, leading to incessant tachycardia; and (3) diagnostic findings during electrophysiology testing, including His-refractory VPDs either delaying the subsequent atrial depolarization or terminating the tachycardia.

## Figures and Tables

**Video 1:** Video of electroanatomic map of atrial activation sequence, demonstrating atrial insertion of the pathway just anterior to the coronary sinus ostium.
